# Validating a multinomial processing tree model for measuring confidence in lineups using a post-response feedback manipulation

**DOI:** 10.1186/s41235-026-00719-9

**Published:** 2026-03-16

**Authors:** Raoul Bell, Nicola Marie Menne, Axel Buchner

**Affiliations:** Department of Experimental Psychology, Heinrich Heine University Düsseldorf, Düsseldorf, Germany

**Keywords:** Eyewitness identification, Lineups, Post-identification feedback, Multinomial processing tree model, Confidence judgements

## Abstract

Confidence in lineup responses is important in research and practice. Here we introduce the lineup confidence model, an extension of the well-validated two-high threshold eyewitness identification model. The two-high threshold eyewitness identification model serves to measure four cognitive processes underlying lineup responses: culprit-presence detection, culprit-absence detection, biased suspect selection and guessing-based selection. The lineup confidence model additionally incorporates the measurement of confidence. To validate the lineup confidence model, we conducted an experiment with a large sample size (*N* = 1565) using post-response feedback as a manipulation of confidence. Confidence followed a predictable and psychologically plausible pattern: responses based on detection were more likely to result in high confidence than responses based on guessing, and responses based on biased suspect selection were also more likely to result in high confidence than responses based on guessing. Importantly, post-response feedback selectively influenced confidence while leaving the parameters for culprit-presence detection, culprit-absence detection, biased suspect selection and guessing-based selection unaffected. Confidence can thus be measured with the model without compromising the measurement of the other processes specified by the model. This successful validation indicates that the lineup confidence model may be useful for examining how lineup characteristics and external factors influence confidence as a function of the processes underlying lineup responses.

## Significance statement

Confidence plays a central role in how legal decision-makers interpret witnesses’ responses to lineups. In addition to making a lineup response (identifying someone or rejecting the lineup), witnesses are often asked to indicate how sure they are, using verbal phrases like “very sure” or numerical ratings like “90 %”. It is recommended in best-practice guidelines to collect confidence judgments immediately after all lineup outcomes, including suspect identifications, filler identifications and rejections. Expressed confidence can strongly influence the weight given to witness testimony. However, confidence can be affected by external factors such as feedback from police officers, highlighting the need to understand how such factors influence confidence. To support this research effort, we developed and validated the lineup confidence model, a formal cognitive model that builds on an existing framework for measuring the processes underlying lineup responses. This model allows examining how confidence varies depending on whether a lineup response is based on culprit-presence and culprit-absence detection, biased suspect selection or guessing. We show that confidence can be validly measured using this model without compromising the measurement of the processes underlying lineup responses. The model thus offers a new measurement tool for examining how lineup procedures and post-response influences affect confidence. This helps researchers and practitioners to better understand the factors that determine when confidence is informative and when it may be misleading.

## Introduction

Lineups are widely used in criminal investigations to gather evidence regarding the guilt or innocence of a suspect. A lineup typically consists of a suspect and several fillers who are known to be innocent (Wells et al., 2020). Ideally, a witness detects the presence or absence of the culprit, leading either to a correct identification of the culprit in a culprit-present lineup or to a correct rejection of a culprit-absent lineup (Menne et al., 2022; Winter et al., 2022). However, witnesses also identify known-to-be-innocent fillers in a surprisingly large number of cases, suggesting that “witness guessing is not restricted to experimental situations with disinterested college students who know that their choices carry no consequences” (Horry et al., 2012). Exonerations of wrongful convictions show that witness identifications can be a major source of error in the justice system, with potentially devastating consequences for innocent suspects (Innocence Project, 2016).

After having identified a lineup member or having rejected the lineup, witnesses are routinely asked how confident they are that their decision is correct. For instance, witnesses may be asked to indicate their confidence through numerical ratings (e.g., “90%”) or verbal labels (e.g., “very sure”). Confidence may provide important information in addition to the lineup response per se and may influence the weight given to the lineup response in legal settings (Brewer & Burke, 2002; Cutler et al., 1988; Wixted & Wells, 2017)*.* For many years, confidence was widely believed to be only weakly related to accuracy (Sporer et al., 1995). However, subsequent theoretical and methodological advances have led to the more optimistic assessment that, when confidence is assessed under pristine conditions—that is, on the initial test, immediately after the lineup decision, using double-blind procedures and fair lineups, without any post-response feedback from the lineup administrator—high confidence is strongly predictive of high accuracy (Wixted & Mickes, 2022; Wixted & Wells, 2017; Wixted et al., 2015, 2018). Signal detection theory inherently predicts a strong confidence-accuracy relationship (Mickes et al., 2012; Wixted et al., 2015). This relationship has enabled the use of receiver operating characteristic analyses, which have led to major empirical advances in the field (for a review, see Mickes et al., 2025). Work grounded in signal detection theory has shown, for instance, that, contrary to longstanding beliefs, simultaneous lineups are diagnostically superior to sequential lineups (Mickes et al., 2012). Moreover, the finding that lineup responses and their associated confidence judgments are most reliable on the initial test has important applied implications: initial lineup responses and confidence judgments, regardless of whether a lineup member is identified or the lineup is rejected, can contain highly diagnostic information, whereas later responses and confidence statements made at trial, often elicited months or years after the crime, are likely to be contaminated. This insight has contributed to the re-evaluation of past cases and to the exoneration of wrongfully convicted individuals who spent many years behind bars (Mickes & Wixted, 2025; Mickes et al., 2025). Collectively, these developments highlight the applied relevance of eyewitness confidence. According to best-practice recommendations, confidence should be collected immediately after the lineup response is made, and it should be collected for all types of lineup responses, including suspect identifications, filler identifications and lineup rejections (Wells et al., 2020). At the same time, the strength of the confidence-accuracy relationship and the precise conditions under which high confidence is predictive of high accuracy continue to be the subject of ongoing debate (e.g., Berkowitz & Frenda, 2018; Fitzgerald et al., 2025; Smalarz, 2021; Smith et al., 2021) and empirical investigation (e.g., Giacona et al., 2021; Lockamyeir et al., 2020).

Against this background, we extend the two-high threshold (2 HT) eyewitness identification model by incorporating the measurement of confidence. The 2 HT eyewitness identification model (Menne et al., 2022; Winter et al., 2022) is a formal measurement model designed to separately measure detection-based and non-detection-based latent processes underlying lineup responses. Specifically, as described in detail below, the model serves to measure culprit-presence detection, culprit-absence detection, biased selection of a lineup member due to lineup unfairness and guessing-based selection as separate latent processes underlying lineup responses. The measurement of these latent processes has proven useful in clarifying how variables such as crime-to-lineup delay (Therre et al., 2025a), pre-lineup instructions (Menne et al., 2025; Winter et al., 2023), position of the suspect in the lineup (Mayer et al., 2024), lineup size (Menne et al., 2023a; Therre et al., 2024), age-related changes (Mayer et al., 2025), verbal overshadowing (Therre et al., 2025b) or filler selection (Bell et al., 2024; Menne et al., 2023b) influence the latent processes underlying lineup responses. Another notable finding is that, when data across multiple experiments are combined to increase statistical sensitivity, a small but consistent advantage of simultaneous over sequential lineups in culprit-presence detection emerges (Winter et al., 2022), paralleling the conclusions derived from signal detection theory regarding discrimination (Mickes et al., 2012). Extending this model to confidence allows examining how confidence relates to each of the detection-based and non-detection-based latent processes of the 2 HT eyewitness identification model.

**Fig. 1:**
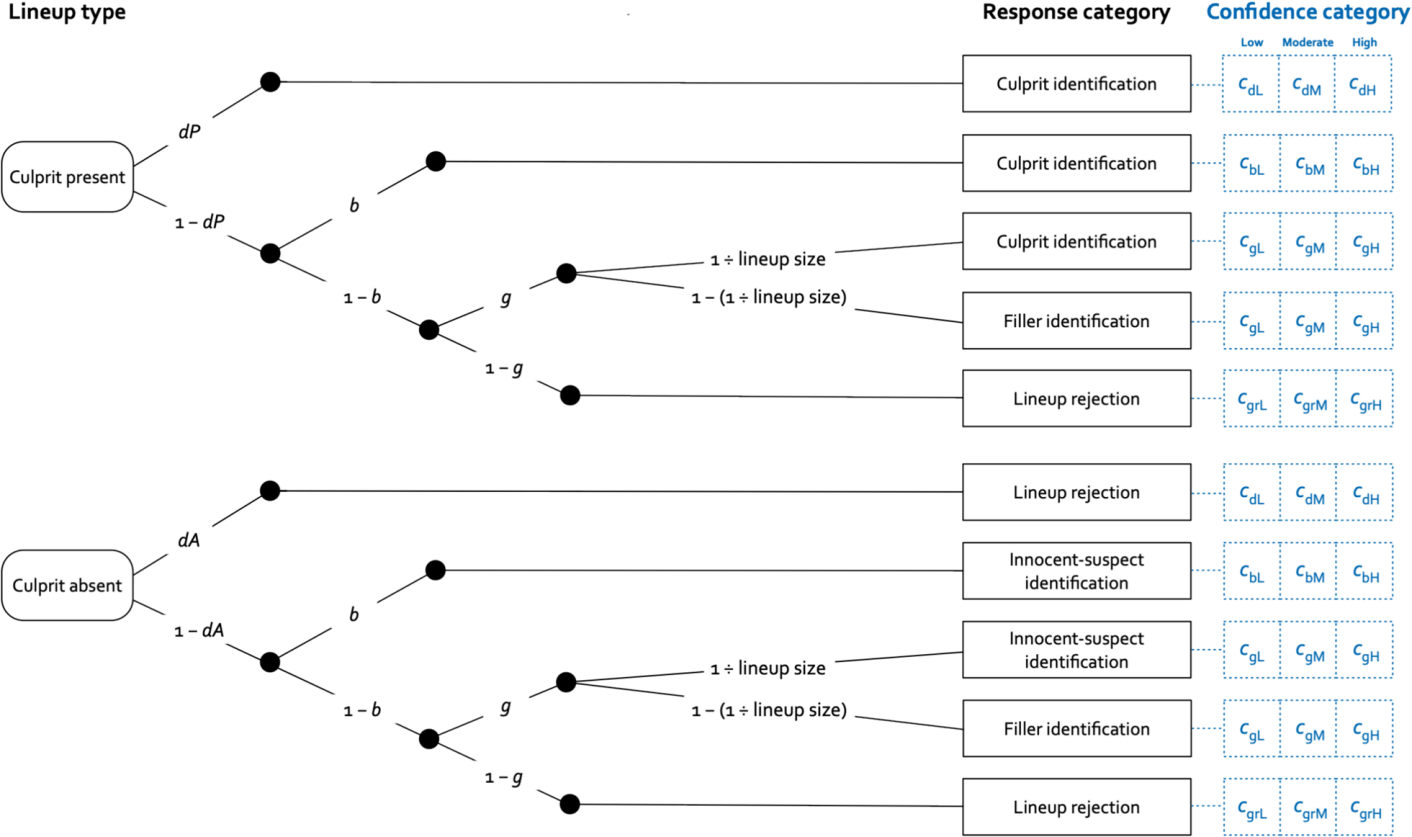
Illustration of the lineup confidence model. *Note.* The 2 HT eyewitness identification model, as originally proposed (Menne et al., 2022; Winter et al., 2022), is illustrated in black. The upper tree refers to culprit-present lineups, the lower tree refers to culprit-absent lineups. Letters along the branches denote the parameters representing the latent processes specified in the model: culprit-presence detection (*dP*), culprit-absence detection (*dA*), biased suspect selection (*b*) and guessing-based selection (*g*). The lineup responses resulting from these processes are represented by black rectangles: culprit identification, innocent-suspect identification, filler identification and lineup rejection. The extension required to incorporate confidence is shown in blue and comprises probabilities of low (*c*_•L_), moderate (*c*_•M_) and high (*c*_•H_) confidence following culprit-presence and culprit-absence detection (*c*_d•_), biased suspect selection (*c*_b•_), guessing-based selection (*c*_g•_) and rejection (*c*_gr_)

The 2 HT eyewitness identification model, as originally introduced (Menne et al., 2022; Winter et al., 2022), is illustrated in black in Fig. 1. This model belongs to the class of multinomial processing tree (MPT) models (Riefer & Batchelder, 1988), which have been widely applied to analyze latent processes underlying categorical behavioral data in domains such as memory (Bayen et al., 1996; Bröder & Meiser, 2007; Meiser, 2014; Smith & Bayen, 2004) and decision making (Erdfelder & Buchner, 1998b; Gawronski et al., 2017; Mieth et al., 2021; Unkelbach & Stahl, 2009). Excellent introductory texts and tutorials make MPT modeling easily accessible to researchers across fields (Erdfelder et al., 2009; Schmidt et al., 2025). Parameter estimation and hypothesis testing can be carried out with freely available software such as multiTree (Moshagen, 2010), MPTinR (Singmann & Kellen, 2013) and TreeBUGS (Heck et al., 2018).

The 2 HT eyewitness identification model provides a formal measurement model for the analysis of lineup data. Any analysis of lineup data necessarily rests on assumptions about how the lineup responses are generated. Ideally, these assumptions are made explicit and tested against the data. In the 2 HT eyewitness identification model, these assumptions are transparently specified in the model’s structure and it can be tested whether these assumptions are compatible with the data. The verbal labels assigned to the model’s parameters serve as accessible descriptors to facilitate communication about the processes specified in the model.

The 2 HT eyewitness identification model accounts for all response categories that can occur in culprit-present and culprit-absent lineups: suspect identifications, filler identifications and lineup rejections in both culprit-present and culprit-absent lineups. From the responses in these categories, the probabilities of the underlying processes are determined: culprit-presence detection (*dP*), culprit-absence detection (*dA*), biased suspect selection (*b*) and guessing-based selection (*g*). Although descriptions of the model are available in previous work (Bell et al., 2024; Mayer et al., 2024; Mayer et al., 2025; Menne et al., 2025; Menne et al., 2022, 2023a, 2023b; Schaper et al., 2025; Therre et al., 2024, 2025a, 2025b; Winter et al., 2022, 2023), the model’s assumptions are fully described here again for convenience and clarity.

In a culprit-present lineup (upper tree in Fig. 1), the presence of the culprit is detected with probability *dP*. Detection of the culprit’s presence may depend, for example, on exposure duration and viewing conditions at encoding (Menne et al., 2022; Winter et al., 2022). With probability 1–*dP*, the culprit is not detected. In this case, the culprit may still be selected through non-detection-based processes. With probability *b*, biased suspect selection occurs, implying that the suspect is selected because the photograph or the face depicted in the photograph stands out from those of the fillers. Parameter *b* thus captures lineup unfairness (Menne et al., 2023b), ensuring that the measurement of the processes represented by the other model parameters is not contaminated by lineup unfairness (Menne et al., 2022; Winter et al., 2022). With probability 1–*b*, no biased suspect selection occurs. However, a lineup member may still be selected on the basis of guessing with probability *g*. Guessing-based selection may be increased, for example, when pre-lineup instructions imply a high compared to a low probability that the culprit is present (Therre et al., 2024; Winter et al., 2022). Guessing is defined as a selection process that is driven neither by detection nor by lineup unfairness, implying no systematic preference for selecting the suspect over the fillers. Consequently, guessing leads to the selection of the suspect with the probability 1 ÷ lineup size and to the selection of a filler with the complementary probability 1–(1 ÷ lineup size). For instance, in a six-person lineup, guessing leads to the selection of the suspect with a probability of 1 ÷ 6 =.17 and to the selection of a filler with a probability of 5 ÷ 6 =.83, illustrating that “a witness who chooses randomly is far more likely to land on a filler than the suspect” (Wixted & Wells, 2017). Importantly, this sampling probability applies specifically to guessing-based selections conditional on no biased suspect selection having occurred, that is, after lineup unfairness has been accounted for through parameter *b*, and therefore does not reflect the overall suspect identification rate. With probability 1–*g*, no lineup member is selected based on guessing, leading to the lineup being rejected.

In a culprit-absent lineup (lower tree in Fig. 1), the absence of the culprit is detected with probability *dA*, leading to a correct lineup rejection. This process is sensitive to manipulations that affect the ease with which a culprit-absent lineup can be rejected (Menne et al., 2022; Winter et al., 2022). With probability 1–*dA*, the absence of the culprit is not detected, in which case the same non-detection-based processes determine lineup responses as in culprit-present lineups. This is so because, in the absence of detection, witnesses do not distinguish between culprit-present and culprit-absent lineups. However, a difference from culprit-present lineups is that biased suspect selection and guessing-based selection lead to the selection of an innocent suspect instead of a culprit.

The 2 HT eyewitness identification model is well validated. When the model was introduced, dedicated validation experiments were conducted with the explicit goal of testing whether the model parameters capture the processes they were designed to measure (Winter et al., 2022). The logic of such validation studies is straightforward. A parameter should be sensitive to manipulations which directly target the process the parameter is intended to measure. Ideally, these manipulations are chosen such that there is a general consensus as to which process they should affect. For instance, pre-lineup instructions that communicate a high versus low probability that the culprit is in the lineup provide an obvious manipulation of guessing processes (Bröder & Schütz, 2009; Buchner et al., 1995). Accordingly, the fact that this manipulation reliably and selectively affects the guessing-based selection parameter *g* (Winter et al., 2022) is evidence of the validity of this parameter. In this way, every parameter of the 2 HT eyewitness identification model has been validated in dedicated validation experiments. In addition, the model has been applied to existing datasets (Menne et al., 2022) that had been published by multiple research groups around the world (Colloff et al., 2016; Karageorge & Zajac, 2011; Lampinen et al., 2020; Malpass & Devine, 1981; Memon et al., 2003; Smith, 2020; Wetmore et al., 2015; Wilcock & Bull, 2010), demonstrating that the validity of the model holds for different materials and procedures.

As it stands, the 2 HT eyewitness identification model does not take confidence into account, but it can be straightforwardly extended to incorporate confidence while preserving its character as a formal measurement model. The extension is shown in blue in Fig. 1. For brevity and to emphasize the broader scope, we henceforth refer to the extended model as the *lineup confidence model*. The lineup confidence model takes into account all confidence judgments following lineup responses, that is, confidence judgments following suspect identifications, filler identifications and lineup rejections in both culprit-present and culprit-absent lineups. Confidence judgments are classified into three confidence categories: low, moderate and high. Confidence probabilities *c*_•L_, *c*_•M_ and *c*_•H_ denote the conditional probabilities of low, moderate and high confidence, respectively, contingent upon the cognitive processes underlying the lineup responses.

Specifically, in culprit-present lineups, the detection of the presence of the culprit results in low confidence with probability *c*_dL_, in moderate confidence with probability *c*_dM_ and in high confidence with probability c_dH_. Biased suspect selection results in low confidence with probability *c*_bL_, in moderate confidence with probability *c*_bM_ and in high confidence with probability *c*_bH_. Guessing-based selection results in low confidence with probability c_gL_, in moderate confidence with probability *c*_gM_ and in high confidence with probability *c*_gH_. Guessing-based rejection results in low confidence with probability *c*_grL_, in moderate confidence with probability *c*_grM_ and in high confidence with probability *c*_grH_. In culprit-absent lineups, detection of the culprit’s absence results in low confidence with probability *c*_dL_, in moderate confidence with probability *c*_dM_ and in high confidence with probability *c*_dH_. Note that detection leads to the same confidence probabilities regardless of whether the culprit’s presence or absence is detected. Because witnesses cannot distinguish between culprit-present and culprit-absent lineups when detection fails, confidence for the non-detection based processes is necessarily the same for culprit-present and culprit-absent lineups. These conditional probabilities for low, moderate and high confidence are not determined directly; rather, they are derived from a binary implementation of the lineup confidence model (see the model equation files at the OSF project page) and then expressed as low, moderate and high confidence. This approach is parallel to how other MPT models, originally designed to measure memory processes, have been extended to incorporate confidence (Bröder et al., 2013; Erdfelder & Buchner, 1998a; Schütz & Bröder, 2011).

As with the original 2 HT eyewitness identification model (Menne et al., 2022; Winter et al., 2022), it is essential to validate the lineup confidence model before applying it to novel research questions. To this end, we conducted a validation experiment with a large sample size to test whether the model parameters capture the psychological constructs they are designed to measure. This validity test has two components.

The first component is a test of whether the confidence probabilities follow a predictable and psychologically plausible pattern. The lineup confidence model is a measurement model in which the confidence probabilities are, in principle, free to adopt any pattern. However, a critical requirement for considering the lineup confidence model a valid measurement instrument is that the confidence probabilities exhibit a pattern consistent with established knowledge about eyewitness confidence. Specifically, responses based on detection should be more likely to yield high confidence than responses based on guessing (Bröder et al., 2013; Erdfelder & Buchner, 1998a; Schütz & Bröder, 2011). Moreover, given that “it is well known that an unfair lineup leads to a higher rate of suspect identification and higher confidence in that identification, whether or not the suspect is the perpetrator” (Wixted & Wells, 2017), biased suspect selection should be more likely to result in high confidence than guessing-based selection or rejection.

The second, and central, component is a test of whether experimental manipulations that are unequivocally expected to affect confidence produce systematic changes in the confidence probabilities. To this end, we chose post-response feedback (Steblay et al., 2014; Wells & Bradfield, 1998) as a manipulation of confidence. Post-response feedback has been shown to robustly influence eyewitness confidence following correct identifications, false identifications and false rejections in culprit-present lineups, as well as false identifications and correct rejections in culprit-absent lineups (Semmler et al., 2004). Specifically, participants received either negative feedback (“This is unexpected. Most people gave a different answer”.) or positive feedback (“You did very well! This matches what most people answered”.), regardless of whether a participant’s lineup response was correct or false and regardless of whether a participant had identified the suspect or a filler or whether a participant had rejected the lineup. Feedback type (negative vs. positive) was manipulated within subjects. Because the study involved a multiple-culprit crime scenario with four culprits, each participant completed four lineups (one for each culprit) and received both negative and positive feedback across the four trials. This design served two purposes. First, it increased the number of data points available for estimating the model parameters with adequate statistical precision. Second, a within-subject manipulation may strengthen the effect of the feedback by making the manipulation more salient than it would otherwise be, which is desirable given the goal of the present research. That goal was not to examine whether positive versus negative feedback affects eyewitness confidence—a finding that is already well established—but to use this well-known effect to test whether the confidence probability estimates of the lineup confidence model sensitively capture this manipulation while the parameters representing the underlying cognitive processes remain unaffected. For this goal, a strong manipulation is advantageous because it provides a particularly powerful test of the sensitivity and selectivity of the model parameters. Specifically, if the lineup confidence model is valid, all confidence probabilities should be sensitive to this manipulation. What is more, the feedback manipulation should selectively affect confidence and leave the other processes specified by the model unaffected. Because feedback is provided only after the lineup response has been made, it is conceptually clear that this post-response feedback cannot retroactively alter the latent cognitive process that produced the response. This makes post-response feedback particularly suitable for testing whether the measurement model functions as intended. If the lineup confidence model is valid, the parameters for culprit-presence detection, culprit-absence detection, biased suspect selection and guessing-based selection must remain unaffected by the feedback manipulation.

## Method

### Participants

Participants were recruited using the ISO-20252-certified research panel of Horizoom (www.horizoom-panel.de). We aimed to collect at least 1500 valid data files and stopped data collection at the end of the day this goal was achieved. Of the 1849 data files from participants who had completed the initial socio-demographic questionnaire, 213 were excluded because participants had not completed the experiment, 26 were excluded because participants had revoked their consent to the use of their data at the end of the experiment, 35 were excluded because participants had seen the staged-crime video more than once and 10 were excluded because participants had failed the attention check (see Materials and procedure section). The final sample consisted of 1565 participants (808 female, 752 male, 5 non-binary) with a mean age of 47 years (*SD* = 16). A sensitivity analysis was conducted using G*Power (Faul et al., 2007). Consistent with previous research (Bell et al., 2024; Mayer et al., 2024; Menne et al., 2025; Menne et al., 2023a, 2023b; Therre et al., 2024, 2025a, 2025b; Winter et al., 2023), the sensitivity analysis was based on a *χ*^2^ goodness-of-fit test given that the goodness-of-fit statistic *G*^2^ used for the model-based statistical test is asymptotically *χ*^2^ distributed (Hu & Batchelder, 1994). The sensitivity analysis showed that, given error probabilities of *α* = *β* =.05 (and thus a statistical power of 1–*β* =.95) and a sample size of *N* = 1565 as well as four lineup responses per participant, it was possible to detect an effect of size *w* =.05 in the critical tests of whether the probabilities of high confidence differ across the negative and positive feedback conditions (*df* = 1).

### Ethics statement

All participants provided informed consent. The ethics committee of the Faculty of Mathematics and Natural Sciences at Heinrich Heine University Düsseldorf has granted approval for a series of experiments comprising the present experiment. At the beginning of the experiment, participants were warned that they would view a short video containing verbal and physical abuse and they were advised to withdraw from the study if they felt uncomfortable viewing such material. At the end of the experiment, participants were informed that the crime depicted in the video had been staged. They were also informed that the feedback they had received during the experiment had been randomly assigned to their responses and could not be used to draw any conclusions about the accuracy of their responses. The experiment was conducted in accordance with the relevant guidelines and regulations.

### Materials and procedure

The experiment was conducted online using SoSci Survey (Leiner, 2019). Participation was restricted to desktop and laptop computers. Participants were instructed to complete the study alone in a distraction-free environment. The same materials and procedure were used as in previous studies (Bell et al., 2024; Mayer et al., 2024; Mayer et al., 2025; Menne et al., 2025; Menne et al., 2023a, 2023b; Schaper et al., 2025; Therre et al., 2024, 2025a, 2025b; Winter et al., 2022, 2023), except for the manipulation of confidence through the post-response feedback manipulation described below. For completeness, we include a full description again here.

#### Staged-crime videos

After having provided sociodemographic information, participants were randomly assigned to one of two staged-crime videos, Video A and Video B. Both videos depicted the same timing and sequence of events: four male culprits, dressed in FC Bayern München fan clothing, verbally and physically assaulting a male victim in Borussia Dortmund fan clothing at a bus station. The culprits’ faces were clearly visible from multiple angles, including frontal views. Different actors were used in the two videos, but each actor in Video A was matched to an actor in Video B in terms of body shape, hair color and hair style. Each video lasted about 130 s and was presented at a resolution of 885 × 500 pixels. After having viewed the staged-crime video, participants answered an attention check question. Out of ten alternatives, they had to correctly identify that the individuals depicted in the video were soccer fans to proceed with the experiment.

#### Lineups

Participants then received the lineup instructions. They were asked to identify the aggressive Bayern München fans from the video. The instructions emphasized that none of the individuals in the lineup might be a culprit. Participants were told to identify someone if they recognized one of the culprits and to otherwise reject the lineup.

Four lineups were presented consecutively, with their order randomized for each participant. Each lineup consisted of one suspect and five fillers, displayed in a single row. The positions of the photographs within each lineup were randomized for each participant. All images were presented at a resolution of 142 × 214 pixels. Examples of the lineups can be found in Winter et al. (2022).

Each participant responded to two culprit-present lineups and two culprit-absent lineups. As in our previous studies (Bell et al., 2024; Mayer et al., 2024; Mayer et al., 2025; Menne et al., 2025; Menne et al., 2023a, 2023b; Schaper et al., 2025; Therre et al., 2024, 2025a, 2025b; Winter et al., 2022, 2023), the crossed-lineup procedure was used. In a culprit-present lineup, the suspect was a culprit from the video participant had previously viewed. In a culprit-absent lineup the suspect was a culprit from the video participant had not viewed. For example, if participants had viewed Video B, two randomly selected culprits from Video B, such as Culprits 1 and 3, served as the culprits in the culprit-present lineups, while Culprits 2 and 4 from Video A served as the innocent suspects in the culprit-absent lineups. The crossed-lineup procedure is similar to the single-lineup procedure proposed by Oriet and Fitzgerald (2018), in which a single lineup is shown to all participants and culprit presence is determined by which of two videos the participants viewed. Following the recommendation of Quigley-McBride and Wells (2021), the crossed-lineup procedure improves on the single-lineup procedure by ensuring that the same person serves as a culprit for some participants and as an innocent suspect for others. This is particularly well suited for the present design because it allows for systematic variation in culprit presence versus absence across multiple lineups and guarantees that no systematic differences can exist between photographs of culprits and innocent suspects. Suspect photographs were taken immediately after the staged-crime videos had been filmed. Filler photographs were selected from the Center for Vital Longevity Face Database (Minear & Park, 2004) to match the suspects in body shape, hair color and hairstyle. Suspect and filler images were standardized for size, lighting, color, and contrast. All photographs depicted a frontal facial view with a neutral expression against a black background, with no visible clothing. Despite these efforts, perfect lineup fairness should not be taken for granted. Rather than presupposing perfect lineup fairness, the crossed-lineup procedure allows unfairness to be measured using the biased-suspect-selection parameter *b*, which has been demonstrated empirically to capture lineup unfairness, ensuring that the measurement of the other processes is not contaminated by lineup unfairness (Menne et al., 2022, 2023b; Winter et al., 2022).

To identify a face, participants clicked a button labeled “Yes, was present” located beneath each photograph. To reject a lineup, participants selected the button labeled “No, none of these persons was present” displayed to the right of the lineup.

#### Post-response feedback

After having clicked the “Next” button, participants received feedback about their response. In the negative-feedback condition, the message “This is unexpected. Most people gave a different answer.” appeared in red. In the positive-feedback condition, the message “You did very well! This matches what most people answered.” appeared in green. Each participant received both types of feedback once for a culprit-present lineup and once for a culprit-absent lineup. The order of the feedback messages and the assignment of the feedback messages to specific lineups were randomized for each participant.

#### Confidence judgment

Below the feedback message, a confidence scale was presented. Participants rated their confidence in their lineup response on a scale from 0% (“not confident”) to 100% (“very confident”). After having provided the confidence judgment, they advanced to the subsequent lineup by clicking the “Next” button. Once all four lineups had been completed, participants were thanked and debriefed.

## Results

Goodness-of-fit tests and parameter estimates were obtained using multiTree (Moshagen, 2010). Confidence judgments were categorized as low (0% to 30%), moderate (31% to 70%) or high (71% to 100%). The observed response frequencies forming the basis for the analyses are reported in Table 1.

**Table 1:** Observed response frequencies (proportions in parentheses) for suspect identifications, filler identifications and lineup rejections in culprit-present and culprit-absent lineups as a function of feedback type (negative, positive) and confidence (low, moderate, high)

	Feedback type					
	Negative feedback			Positive feedback		
	Confidence					
	Low	Moderate	High	Low	Moderate	High
	Culprit-present lineups					
Culprit identifications	142 (.09)	234 (.15)	242 (.15)	53 (.03)	195 (.12)	375 (.24)
Filler identifications	180 (.12)	246 (.16)	48 (.03)	92 (.06)	227 (.15)	164 (.10)
False lineup rejections	213 (.14)	187 (.12)	73 (.05)	95 (.06)	164 (.10)	200 (.13)
	Culprit-absent lineups					
Innocent-suspect identifications	65 (.04)	97 (.06)	35 (.02)	24 (.02)	87 (.06)	109 (.07)
Filler identifications	253 (.16)	305 (.19)	69 (.04)	109 (.07)	301 (.19)	224 (.14)
Correct lineup rejections	287 (.18)	323 (.21)	131 (.08)	128 (.08)	256 (.16)	327 (.21)

To analyze these data, two instances of the lineup confidence model displayed in Fig. 1 were needed, one for the negative-feedback condition and one for the positive-feedback condition. Given that six-person lineups were used, the constant 1 ÷ lineup size was set to .16667 as an approximation of ⅙. To maintain consistency with previous applications of the 2 HT eyewitness identification model (Menne et al., 2025; Menne et al., 2022, 2023a; Therre et al., 2024, 2025b; Winter et al., 2022, 2023), we also constrained the biased-suspect-selection parameter *b* and the culprit-absence detection parameter *dA* to be equal across the conditions.^1^ The resulting base model fit the data, *G*^2^(10) = 6.54, *p* =.768, indicating that these constraints are compatible with the data. To check for identifiability, we used multiTree’s (Moshagen, 2010) repeated analysis module with 1000 repetitions. Identical parameter estimates were obtained in all cases, suggesting that the model is identifiable. Parameter *b* was estimated to be .06 (*SE* =.01). This value is slightly above zero, indicating that the lineups were not perfectly fair. However, this does not pose a problem for any of the model-based analyses because the biased-suspect-selection parameter *b* absorbs lineup unfairness, ensuring that all other model parameters are measured in an uncontaminated way (Menne et al., 2022, 2023b; Winter et al., 2022). For the present research question, some degree of lineup unfairness is actually beneficial because it enables measurement of confidence following biased suspect selection. Parameter *dA* was estimated to be .06 (*SE* =.02). Parameter estimates for culprit-presence detection (*dP*) and guessing-based selection (*g*) are reported separately for the two feedback conditions in Table 2.

**Table 2:** Estimates of the culprit-presence-detection parameter *dP* and the guessing-based selection parameter *g* as a function of feedback type (negative, positive)

	Negative	Positive
Parameter *dP*	.30 (.02)	.30 (.02)
Parameter *g*	.54 (.01)	.56 (.01)

Values in parentheses represent standard errors.

**Fig. 2:**
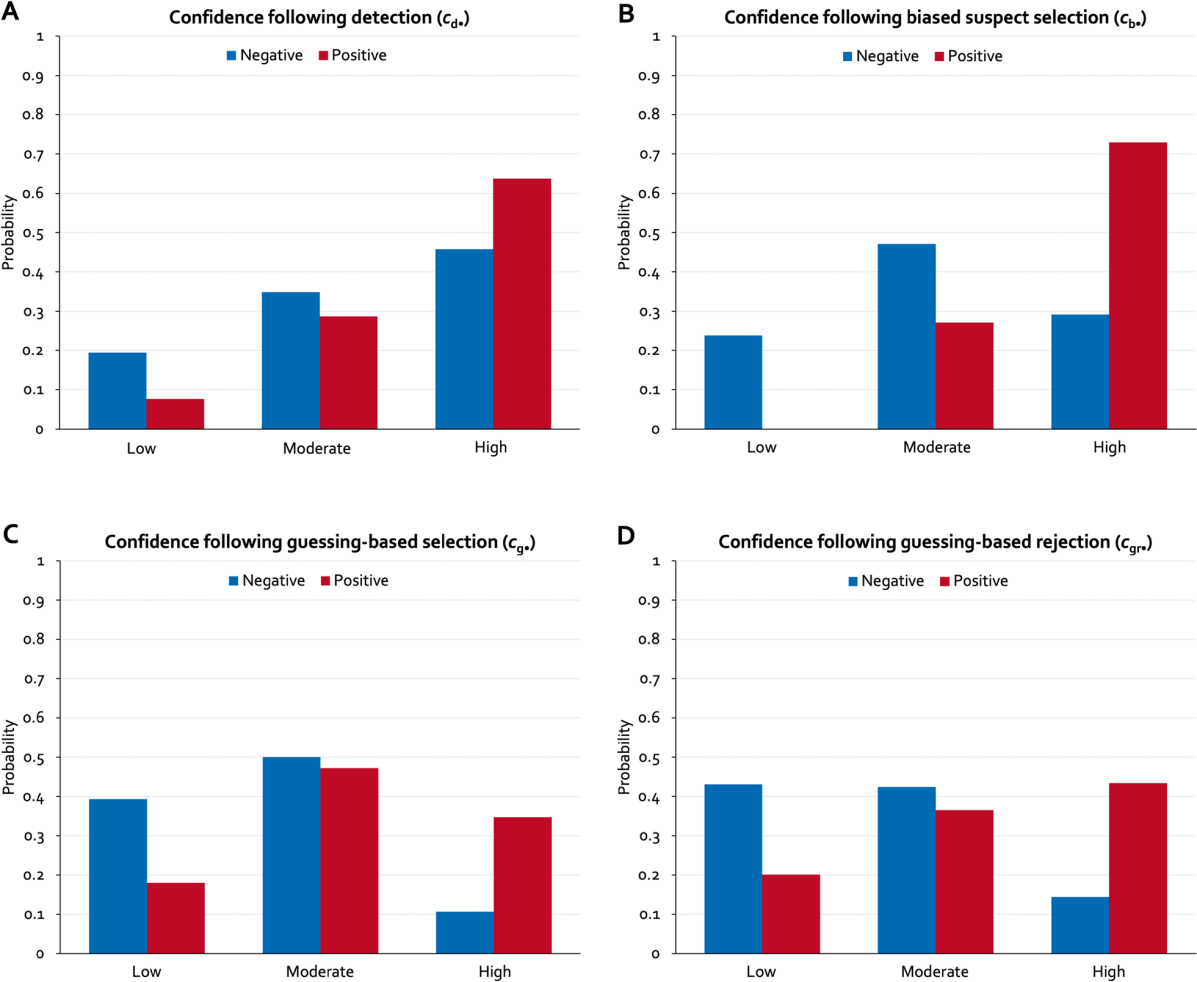
Probability estimates of low, moderate and high confidence following culprit-presence and culprit-absence detection (Panel A), biased suspect selection (Panel B), guessing-based selection (Panel C) and guessing-based rejection (Panel D) as a function of feedback type (negative, positive). *Note.* To facilitate communication and interpretation, the parameter estimates obtained within the binary implementation of the processing tree model (see the model equation files at the OSF project page) were transformed into probability estimates of low, moderate and high confidence, separately for confidence following detection, biased suspect selection, guessing-based selection and rejection (see the spreadsheet file at the OSF project page)

Interpretation and communication with respect to confidence are facilitated when the confidence-related parameter estimates obtained within the binary implementation of the processing tree model (see the model equation files at the OSF project page) are transformed into probability estimates of low, moderate and high confidence. This was done separately for confidence following detection, biased suspect selection, guessing-based selection and rejection (see the spreadsheet file at the OSF project page). The resulting confidence probability estimates are displayed in Fig. 2 as a function of feedback type.

We first compared confidence as a function of the process underlying the response to test whether the confidence probabilities followed a psychologically plausible pattern. As expected, detection (Panel A) was more likely to result in high confidence than either guessing-based selection (Panel C) or guessing-based rejection (Panel D). These descriptive differences were statistically significant. High confidence was significantly more likely following detection than guessing-based selection, ∆*G*^2^(2) = 221.21, *p* <.001, and guessing-based rejection, ∆*G*^2^(2) = 135.73, *p* <.001. Notably, the results also showed that high confidence was significantly more likely following biased suspect selection (Panel B) than following guessing-based selection (Panel C), ∆*G*^2^(2) = 24.62, *p* <.001, and guessing-based rejection (Panel D), ∆*G*^2^(2) = 15.65, *p* <.001.

Second, we tested whether the confidence probabilities sensitively reflected the manipulation of confidence through post-response feedback. As expected, negative feedback was less likely to result in high confidence than positive feedback in all instances. These descriptive differences were statistically significant. High confidence was significantly increased in the positive-feedback condition compared to the negative-feedback condition following detection, ∆*G*^2^(1) = 19.36, *p* <.001, biased suspect selection, ∆*G*^2^(1) = 13.46, *p* <.001, guessing-based selection, ∆*G*^2^(1) = 192.09, *p* <.001, and guessing-based rejection, ∆*G*^2^(1) = 184.36, *p* <.001. These findings demonstrate that the manipulation of confidence through post-response feedback was sensitively captured in the confidence probabilities.

Because the feedback was presented only after participants had given their lineup response, it necessarily follows that, if the measurement model is valid and functions as intended, the feedback manipulation should selectively affect the confidence probabilities while leaving the parameters representing culprit-presence detection, culprit-absence detection, biased suspect selection and guessing-based selection unaffected. Consistent with this expectation, the feedback manipulation had no effect on the culprit-presence-detection parameter *dP,* ∆*G*^2^(1) < 0.01, *p* =.951, and it also had no effect on the guessing-based selection parameter *g,* ∆*G*^2^(1) = 1.19, *p* =.275. Together with the fact that the biased-suspect-selection parameter *b* and the culprit-absence-detection parameter *dA* were equated across feedback conditions in the base model, which fit the data, these results show that the effects of feedback were selectively reflected in the confidence probabilities, leaving the other parameters of the lineup confidence model unaffected.

## Discussion

Since its introduction (Menne et al., 2022; Winter et al., 2022), the 2 HT eyewitness identification model has proven useful in lineup research for disentangling four cognitive processes underlying lineup responses: culprit-presence detection, culprit-absence detection, biased suspect selection and guessing-based selection (Bell et al., 2024; Mayer et al., 2024; Mayer et al., 2025; Menne et al., 2025; Menne et al., 2023a, 2023b; Schaper et al., 2025; Therre et al., 2024, 2025a, 2025b; Winter et al., 2023). However, the 2 HT eyewitness identification model does not account for confidence. This represents a limitation because confidence may provide important supplementary information in addition to the lineup response per se and may influence the weight given to the lineup response in legal settings (Brewer & Burke, 2002; Cutler et al., 1988; Wixted & Wells, 2017). Here we have introduced the lineup confidence model, an extension of the 2 HT eyewitness identification model that incorporates measures of confidence in addition to measures of culprit-presence detection, biased suspect selection, guessing-based selection and culprit absence-detection. Specifically, we have shown how the 2 HT eyewitness identification model can be straightforwardly extended to include conditional probabilities of low, moderate and high confidence, contingent upon the cognitive processes underlying lineup responses.

In addition, we have reported a validation study, which is an essential initial step before a measurement model should be applied to novel research questions. This validation of the lineup confidence model was successful. As a precondition, the lineup confidence model fit the data, demonstrating that the assumptions of the model were compatible with the observed response frequencies, even under the high statistical sensitivity afforded by the large sample size.

In the first component of the test of validity, the confidence probabilities have been shown to follow a predictable and psychologically plausible pattern. Specifically, responses based on detection should be more likely to yield high confidence than responses based on guessing (Bröder et al., 2013; Erdfelder & Buchner, 1998a; Schütz & Bröder, 2011). This prediction was confirmed. High confidence was increased following detection in comparison to guessing-based selection or rejection. Moreover, given that unfair lineups not only increase false identifications but also enhance the confidence placed in these identifications (Wixted & Wells, 2017), biased suspect selection was predicted to be more likely to result in high confidence than guessing-based selection or rejection. This prediction was confirmed as well: biased suspect selection was more likely to result in high confidence than guessing-based selection and rejection. Taken together, the findings demonstrate that the confidence probabilities of the lineup confidence model follow a predictable and psychologically plausible pattern, providing support for the conclusion that the lineup confidence model is valid.

The second, and central, component of model validation was the test of whether an experimental manipulation that is unequivocally expected to affect confidence produces systematic changes in the confidence probabilities. For this purpose, a manipulation of post-response feedback (Steblay et al., 2014; Wells & Bradfield, 1998) is ideal because of its well-established and conceptually clear relationship to confidence. Participants received either negative feedback (“This is unexpected. Most people gave a different answer.”) or positive feedback (“You did very well! This matches what most people answered.”), regardless of whether their response was correct or false and regardless of whether they had identified the suspect or a filler or whether they had rejected the lineup. This type of feedback has been shown to exert robust effects on confidence across a wide range of lineup outcomes, including correct identifications, false identifications and false rejections in culprit-present lineups, as well as false identifications and correct rejections in culprit-absent lineups (Semmler et al., 2004). If the lineup confidence model is valid, all confidence probabilities should be sensitive to this manipulation. The results clearly confirm this prediction: post-response feedback significantly increased the likelihood of high confidence following detection, biased suspect selection, guessing-based selection and guessing-based rejection. In contrast, the parameters for culprit-presence detection, culprit-absence detection, biased suspect selection and guessing-based selection were unaffected by the feedback manipulation. The confidence probabilities thus sensitively reflected the post-response feedback manipulation, while the parameters for the cognitive processes underlying the lineup responses remained uncontaminated by the changes in confidence produced by the post-response feedback, providing empirical support for the validity of the lineup confidence model.

The present results already demonstrate the utility of the lineup confidence model for providing a deeper examination of factors that determine confidence. Specifically, the strong influence of post-response feedback confirms that confidence is not solely determined by the processes that generate the response but is also shaped by external cues. Particularly problematic are increases in confidence for non-detection-based processes. In fact, it has been observed that external cues such as post-response feedback affect confidence of inaccurate witnesses more than that of accurate witnesses (Bradfield et al., 2002; Charman et al., 2010; Steblay et al., 2014). One interpretation of this finding is that external cues such as post-response feedback play a larger role when witnesses cannot rely on an internal memory cue. Therefore, it can be predicted that high confidence following detection (*c*_dH_) is less affected by post-response feedback than high confidence following guessing (*c*_gH_ and *c*_grH_). Looking at the high-confidence probability estimates displayed in Fig. 2 (Panel A versus Panels C and D), this is indeed the case at the descriptive level. To test this prediction statistically, we reparametrized the lineup confidence model by implementing parametric order constraints in multiTree (for technical details, see Kuhlmann et al., 2019). In this approach, so-called shrinkage parameters are introduced such that the dimensionality of the model and thus the number of parameters and the degrees of freedom of the test of model fit remain unchanged. The reparameterized model fits the data as well as the original lineup confidence model. In the present case, each parameter representing high confidence under negative feedback was removed from the model and instead re-expressed, via a shrinkage parameter, as a proportion of its corresponding parameter under positive feedback (see the model equation files at the OSF project page). Thus, the smaller the shrinkage parameter, the more pronounced is the reduction in high confidence in the negative-feedback condition relative to the positive-feedback condition. A benefit of the reparameterized version of the model is that the shrinkage parameters can be compared to each other statistically. As predicted, the shrinkage parameter was significantly larger for high confidence following detection (.72, *SE* =.05) than for high confidence following guessing-based selection (.24, *SE* =.02), ∆*G*^2^(1) = 73.77, *p* <.001, and guessing-based rejection (.33, *SE* =.03), ∆*G*^2^(1) = 38.69, *p* <.001. Thus, compared to the positive-feedback condition, high confidence in the negative-feedback condition was relatively preserved when responses were based on detection compared to when responses were based on guessing. This confirms the prediction derived from previous findings (Bradfield et al., 2002; Charman et al., 2010; Steblay et al., 2014) that confidence following detection is less affected by post-response feedback than confidence following guessing. Importantly, the lineup confidence model makes it possible to elegantly test such hypotheses directly at the process level rather than indirectly at the level of observed behavior, thereby ensuring a closer correspondence between the assumptions implied by theorizing about witness confidence and the statistical tests of these assumptions.

Now that the lineup confidence model has successfully passed an important validation test, it can be applied to address new research questions. This includes examining potential effects of manipulations that directly target confidence, such as social pressure to report a certain level of confidence (Bruer et al., 2024), reflecting on cues upon which confidence may be based (Robinson & Johnson, 1998), suggestive questioning about confidence (Greenspan & Loftus, 2020) and co-witness information provided after the lineup response (Luus & Wells, 1994). At the same time, the model makes it possible to disentangle more complex cases in which manipulations may influence not only confidence but also the processes of culprit-presence detection, culprit-absence detection, biased suspect selection and guessing-based selection. Examples include differences in encoding conditions (Lindsay et al., 1998), repeated identification procedures (Godfrey & Clark, 2010) and blind lineup administration (Charman & Quiroz, 2016). Finally, the lineup confidence model may shed light on the observation that the confidence-accuracy relationship is stronger for identifications than for lineup rejections (Sporer et al., 1995). With the lineup confidence model in mind, this asymmetry in the confidence-accuracy relationship at the level of the observable responses can be accounted for based on two findings: First, high confidence is more likely following detection than following guessing (Fig. 2, Panel A vs. Panels C and D). Second, culprit-presence detection occurs with higher probability than culprit-absence detection. As a result, suspect identifications are more likely than lineup rejections to be based on detection. Consequently, high confidence is more diagnostic of detection—and thus accuracy—for suspect identifications than for lineup rejections, producing the observed asymmetry in the confidence-accuracy relationship without requiring separate confidence mechanisms for culprit-presence versus culprit-absence detection.

As with any research, there are some limitations. First, extending the model to measure confidence increases the number of parameters to be estimated. This requires sufficiently large datasets, which may pose challenges in smaller-scale laboratory experiments or applied settings. Second, in the lineup confidence model applied here, confidence was divided into three levels corresponding to low, medium and high confidence. This categorization was chosen to keep the model simple and to ensure that enough data points were available per category, thereby enhancing both the precision of the parameter estimates and the sensitivity of the statistical tests. Future research could explore whether a finer categorization provides additional insights. Third, the lineup confidence model was evaluated using the same stimulus material and procedure already used in studies applying the original 2 HT eyewitness identification model (Bell et al., 2024; Mayer et al., 2024; Mayer et al., 2025; Menne et al., 2025; Menne et al., 2023a, 2023b; Schaper et al., 2025; Therre et al., 2024, 2025a, 2025b; Winter et al., 2022, 2023). Given that the validity of the original 2 HT eyewitness identification model has been established in reanalyses (Menne et al., 2022) of existing datasets previously published by different research groups around the world (Colloff et al., 2016; Karageorge & Zajac, 2011; Lampinen et al., 2020; Malpass & Devine, 1981; Memon et al., 2003; Smith, 2020; Wetmore et al., 2015; Wilcock & Bull, 2010) that had used a variety of materials and procedures, it seems attractive to similarly extend the variety of materials and procedures to which the lineup confidence model is applied. Fourth, post-response feedback was manipulated within subjects, with each participant responding to four lineups and receiving both negative and positive feedback across trials. This design was primarily chosen to increase the number of data points available for estimating the model parameters with adequate statistical precision. It seems possible that feedback received on earlier trials may have influenced responding on later trials (cf. Palmer et al., 2010). For instance, negative feedback on earlier trials may have strengthened the impact of positive feedback on later trials and vice versa. Such trial-to-trial influences remain to be addressed in future experiments specifically designed to examine such effects, which would require much larger sample sizes than the present experiment. Finally, it is important to note the scope of the lineup confidence model: it is a measurement model. Its purpose is to measure the contributions of culprit-presence detection, culprit-absence detection, biased suspect selection and guessing-based selection, along with confidence. It is not intended to address questions such as at what level of confidence an identification or rejection should be considered probative of a suspect’s guilt or innocence. Other approaches have been developed to address such questions (Mickes, 2015; Sauer et al., 2019; Smith et al., 2021; Wixted & Wells, 2017).

## Conclusion

To conclude, we introduced the lineup confidence model, which provides a formal way to measure confidence in the context of lineups, conditional on the cognitive processes that generated the lineup response. We also presented the results of a validation study. These results indicate, first, that the confidence probabilities follow a psychologically plausible and unequivocally predictable pattern and, second, that the confidence probabilities are sensitive to an experimental manipulation of confidence through post-response feedback. This successful validation indicates that the lineup confidence model may serve as a useful tool for examining how lineup characteristics and external factors influence confidence as a function of the processes underlying lineup responses.

## Data Availability

The experiment was not preregistered. The frequency data and model equation files of all analyses as well as trial-level raw data and a spreadsheet file explicating how the confidence probability estimates were computed based on confidence parameter estimates have been made publicly available at the Open Science Framework and can be accessed at https://osf.io/nxj7u

## Footnotes

1 Given that the feedback manipulation occurred after the cognitive processes underlying the lineup responses, it would also have been possible to start with a base model in which culprit-presence detection, culprit-absence detection, biased suspect selection and guessing-based selection are all constrained to be equal across the feedback conditions, which also fits the data. When using this base model, all of the conclusions drawn from the statistical tests reported here remain the same. Alternatively, it would also have been possible to start with a base model without constraining parameters *b* and *dA* to be equal across feedback conditions, which also fits the data. Again, when using this base model, all of the conclusions drawn from the statistical tests reported here remain the same, including the conclusions that *b* and *dA* do not differ across feedback conditions. Thus, the conclusions do not depend on the specific choice of the base model.
